# The comparison of anti-seizure and tocolytic effects of phenytoin and magnesium sulphate in the treatment of eclampsia and preeclampsia: A randomised clinical trial

**Published:** 2017-07-06

**Authors:** Maryam Khooshideh, Majid Ghaffarpour, Sama Bitarafan

**Affiliations:** 1 Department of Obstetrics and Gynecology, School of Medicine, Arash Hospital, Tehran University of Medical Sciences, Tehran, Iran; 2 Iranian Center of Neurological Research, Neuroscience Institute, Tehran University of Medical Sciences, Tehran, Iran

**Keywords:** Phenytoin, Magnesium Sulphate, Cesarean Section, Eclampsia, Preeclampsia

## Abstract

**Background:** To date, magnesium sulphate (MgSO_4_) is the treatment of choice for prevention of seizure in eclampsia and preeclampsia. However, there are some limitations in the administration of MgSO_4_ due to its tocolytic effects. The aim of this study was to compare the anticonvulsant and tocolytic effects of MgSO_4 _and another drug, phenytoin, in patients with eclampsia and preeclampsia.

**Methods:** This clinical trial was conducted on pregnant women hospitalised with eclampsia or preeclampsia, during 2014–2016. The subjects were randomly assigned to two treatment groups using blocking method based on disease (eclampsia or mild and severe preeclampsia). One group received MgSO_4_ (group M) and another group received phenytoin (group P) as treatment. Each group consisted of 110 and 65 women with mild and severe preeclampsia, respectively (subgroup A), and 25 women with eclampsia (subgroup B). Duration of labor, the number of cesarean sections, convulsions and Apgar scores of infants were compared between the two groups and were considered as treatment outcomes.

**Results:** Convulsion rate was significantly lower with MgSO_4_ than phenytoin (P = 0.001). No seizure occurred in patients with mild preeclampsia in group P. Duration of stage one of labor (P < 0.001) and the number of cesarean sections (P = 0.040) were significantly higher in group M. However, one-minute Apgar scores for newborns were higher in women treated with MgSO_4_ compared to that of phenytoin (P = 0.001). Five-minute Apgar was not significantly different.

**Conclusion:** Although MgSO_4_ is more effective than phenytoin for prevention of convulsion in eclampsia and severe preeclampsia, phenytoin may be considered for treatment of special conditions such as mild preeclampsia. Due to the tocolytic effects of MgSO_4_ on increasing the duration of labor, the increased risk of cesarean section and the potential for toxicity, physicians should critically consider the best drug according to the condition of the patient.

## Introduction

Preeclampsia is a prevalent multisystem disorder. It is associated with systolic blood pressure of ≥ 140 mmHg and/or diastolic blood pressure of ≥ 90 mmHg after 20 weeks of gestation and occurrence of proteinuria in previously normotensive patients. Severe preeclampsia can damage several organs such as liver, kidneys, clotting system and brain.^1^^-^^3^ Preeclampsia and eclampsia are the second largest cause of maternal morbidity.^4^^-^^6^

Severe preeclampsia without anti-seizure prophylaxis transforms to eclampsia and new-onset generalised seizures occur in eclamptic women.^7^ Women with eclampsia should receive anticonvulsant therapy but there is controversy about this choice.^8^^-^^10^ Anticonvulsant drugs generally used as prophylaxis are diazepam, phenytoin and MgSO_4,_^7^^,^^10^ but many studies reported that MgSO_4_ is the first choice of therapy.^11^^-^^13^

Convulsion may occur due to interference in the regulation of cerebral circulation, dysfunction of endothelium and brain edema.^7^ MgSO_4_ stimulates the release of prostacyclin from endothelium and acts as a vasodilator, reducing systemic blood pressure and protecting from cerebral edema.^14^^-^^16^

Advantages of MgSO_4_ therapy are rapid onset of action, easily available antidote (calcium gluconate), lack of sedation and low cost. On the other side, some side effects of MgSO_4_ can occur in patients such as painful intramuscular administration, flushing and warmth, nausea, vomiting, headache and muscle weakness. Also, dyspnea, chest pain, pulmonary edema, cardiac arrest and respiratory depression due to magnesium toxicity may be seen in MgSO_4_ therapy.^17^ The tocolytic activity of MgSO_4_ can increase the duration of labor, the number of cesarean section, and post-partum hemorrhage.^9^^,^^18^^,^^19^

However, some traditional anticonvulsant drugs such as phenytoin are useful as alternatives.^8^^,^^9^ Phenytoin crosses the blood-brain barrier rapidly. Side effects of phenytoin include dysrhythmia, hypotension fever, skin rash around the eyes and allergy.^5^ The aim of this study was to compare the effects of MgSO_4_ and phenytoin in terms of method and duration of labor and the rate of seizure in patients with eclampsia and preeclampsia.

## Materials and Methods

After approval from the ethics committee, the present randomised clinical trial study was conducted on 400 pregnant women admitted with eclampsia, mild and severe preeclampsia to Arash Hospital in Tehran, Iran, during the time period 2014-2016 (clinical trial number: IRCT2016120311020N7). 

**Figure 1:**
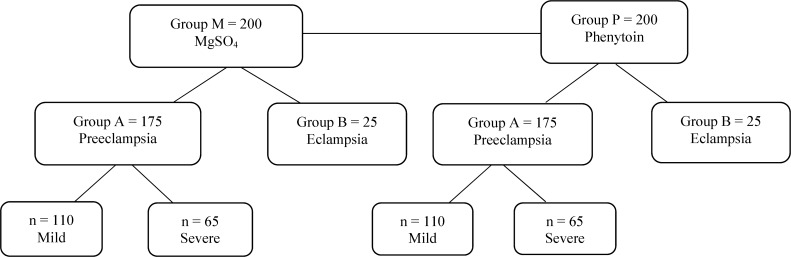
Diagram of the study

Primiparous women with mild and severe preeclampsia and eclampsia and ≥ 34-week gestational age were included in the trial. Patients with heart disease, multifetal pregnancy, smokers, drug users and women with hypersensitivity to MgSO_4_ or phenytoin were excluded. According to the mentioned inclusion criteria, the patients were chosen with random blocking method based on the disease (eclampsia or mild and severe preeclampsia) and were assigned to one of the two treatment groups. One group received MgSO_4_ (group M) and another group received phenytoin (group P) as treatment. In each group, 110 women with mild and 65 women with severe preeclampsia (subgroup A), and 25 women with eclampsia (subgroup B) were included (Figure 1). 

Study outcomes, duration of labor, the number of cesarean sections, the number of convulsion events and Apgar scores of infants were compared between groups.

Mild preeclampsia was defined as systolic blood pressure of ≥ 140 mmHg and/or diastolic blood pressure of ≥ 90 mmHg after 20 weeks of gestation and proteinuria. Severe preeclampsia was defined as systolic blood pressure of ≥ 160 mmHg and/or diastolic blood pressure of ≥ 110 mmHg or when patients with preeclampsia had organ damage such as in liver, kidneys, clotting system, or brain. Eclampsia referred to the occurrence of new-onset tonic-clonic seizures or coma in a patient with preeclampsia. 

Group M received the loading dose of 4 g 20% MgSO_4_ in 100 ml of lactated Ringer's solution intravenously over 20 min, followed by 2 g/hour IV infusion. Patients were monitored every 4 hours regarding the following parameters: presentation of patellar reflex, urine output > 100 ml/4 hours or > 25 ml/hour, and respiratory rate > 12/min. In group P loading dose of 20 mg/kg body weight (maximum 1000 mg) of phenytoin in 100 ml of lactated Ringer's solution was given intravenously slowly over 20 minutes. A maintenance dose of 500 mg was given orally after 10 hours. Therapy was continued for 24 hours post-partum or after the last convulsion following delivery.

The number of convulsions was recorded every four hours and all other laboratory data were recorded every six hours. Complications such as acute renal failure, cerebrovascular accident, and hepatic failure, were also recorded if present. Apgar scores of the fetus at one and five minutes were noted. Duration of labor and the type of delivery were also recorded.

All data analyses were conducted using SPSS for windows (version 16, SPSS Inc., Chicago, IL, USA). Descriptive statistics for continuous variables were presented as mean ± standard deviation and for categorical variables as numbers (percentage). The baseline characteristics of the two groups were compared using an independent t-test for continuous variables and the chi-square test for categorical variables. Moreover, the convulsions were compared between the groups using chi-square test. All statistical tests were two-sided and the level of statistical significance cut-off was set at 0.05. All analyses were performed on an intent-to-treat basis. The conduct and analysis of the trial strictly adhered to the 2010 CONSORT guidelines.

## Results

The mean age of patients in the group M was 27.4 ± 5.9 and in the group P was 27.5 ± 5.7. In the group M, the mean body mass index (BMI) was 24.38 ± 2.72 and in group P it was 24.68 ± 2.74. The differences between the two groups in mean age (P = 0.920) (Table 1) and mean BMI (P = 0.270) (Table 1) were not significant. Also, there was no statistically significant difference between group M (36.25 ± 1.18) and group P (36.45 ± 1.07) in mean gestational age (P = 0.120) (Table 1). 

The number of convulsion attacks in patients allocated in MgSO_4_ group was 0, and in patients treated with phenytoin was 10 (seven convulsions in patients with severe preeclampsia and three convulsions in patients with eclampsia). MgSO_4_ was significantly more efficient than phenytoin for convulsion prevention (P = 0.001) (Table 2). 

Duration of stage 1 of labor was significantly longer in group M (294.4 ± 103.2 min) compared to group P (258.80 ± 101.01 min, P < 0.001) (Table 2). 

Duration of stage 2 of labor was also longer in group M (48.18 ± 16.05 min) compared to group P (46.21 ± 15.20 min), but the differences were not statistically significant (P = 0.200) (Table 2). The rate of cesarean section was significantly greater in group M (45%) compared to group P (35%) (P = 0.040). MgSO_4_ induced longer labor and increased the rate of cesarean sections in comparison to phenytoin. 

There was a statistically significant difference in one-minute Apgar score between group M (8.57 ± 1.50) and group P (8.06 ± 1.66) (P = 0.001) (Table 2) but there was no significant difference in the five-minutes Apgar score (P = 0.340) (Table 2). The one-minute Apgar score for newborns was higher in group M but finally, it was same in both groups.

**Table 1:** Baseline characteristics

**Baseline demographics**	**Group M (n = 200)**	**Group P (n = 200)**	**P**
Age (years) (mean ± SD)	27.40 ± 5.90	27.50 ± 5.70	0.924
Body mass index (kg/m^2^) (mean ± SD)	24.38 ± 2.72	24.68 ± 2.74	0.273
Gestational age (weeks) (mean ± SD)	36.25 ± 1.18	36.45 ± 1.07	0.120

SD: Standard deviation

**Table 2:** Primary and secondary outcomes of study

**Outcomes**	**Group M (n = 200)**	**Group P (n = 200)**	**P**
Apgar 1 min (mean ± SD)	8.57 ± 1.50	8.06 ± 1.66	0.001
Apgar 5 min (mean ± SD)	9.88 ± 0.40	9.86 ± 0.43	0.340
Duration of stage 1 of labor (min) (mean ± SD)	294.40 ± 103.20	258.80 ± 101.01	< 0.001
Duration of stage 2 of labor (min) (mean ± SD)	48.18 ± 16.05	46.21 ± 15.20	0.200
Rate of cesarean section (%)	45	35	0.040
Convulsion in patients with mild preeclampsia (n)	0	0	0.001
Convulsion in patients with severe preeclampsia (n)	0	7	
Convulsion in patients with eclampsia (n)	0	3	

## Discussion

In the present study, seizures were observed in 10 patients in phenytoin group (7 patients in severe preeclampsia subgroup and 3 patients in eclampsia subgroup) and no seizure occurred in MgSO_4_ group and/or in patients with mild preeclampsia in the phenytoin group after the trial. 

MgSO_4_ is known as a better choice compared to phenytoin for prevention of seizures in patients with eclampsia and preeclampsia.^16^^,^^20^ However, Slater and colleagues reported 100% treatment success in their study on 26 women with preeclampsia and eclampsia who were given phenytoin.^21^ Possible explanations for this inconsistency in studies may be the administration of different doses of phenytoin. In a study by Robson, et al., three women had seizures despite receiving therapeutic levels of phenytoin.^22^ Appleton, et al. explained that therapeutic threshold of phenytoin in non-pregnant patients and pregnant women with preeclampsia may be different.^23^

In the present study, both the rate of cesarean section and duration of labor in MgSO_4_ group were significantly higher than phenytoin group. Our study showed that the tocolytic effects of MgSO_4_ are considerably higher compared to that of phenytoin, but MgSO_4_ is also more effective than phenytoin in the prevention of convulsions. 

Our results are in accordance with many previous researches that reported a statistically significant increase in the duration of labor and the rate of cesarean section in patients treated with MgSO_4_ compared to phenytoin.^5^^,^^24^^,^^25^ In contrast, Belfort, et al. showed that MgSO_4_ is a weak tocolytic drug and labor duration does not appear to be affected by its administration.^26^ Moreover, some studies, reported no tocolytic effects for MgSO_4_ in women with preeclampsia.^18^^,^^27^^,^^28^

Phenytoin induced more rapid cervical dilation than MgSO_4_ and did not increase the duration of labor.^25^ Patients treated with phenytoin had significantly less postpartum hemorrhage,^5^^,^^29^ and less time was required to regain consciousness compared to patients treated with MgSO_4_.^5^

Therefore, phenytoin can decrease the need for skilled workers in the delivery room. It might be important for hospitals in the low- and middle-income countries with a high prevalence of patients with preeclampsia and eclampsia and insufficient facilities. Phenytoin can be helpful for hospitals that experience increased bed turn-over in those countries. 

Because of the intensive care requirement for maternal monitoring during intravenous infusion of MgSO_4_ or risk of toxicity, particularly where the capacity of maternal monitoring is limited, phenytoin seems to have practical implications. 

The present study showed that one-minute Apgar score was lower in phenytoin group but five-minute Apgar score did not differ significantly. Roy, et al. showed that infants born in MgSO_4_ group had higher Apgar scores compared to phenytoin group, but the results were not statistically significant.^5^ Phenytoin does not have a significant effect on Apgar score.

## Conclusion

Although MgSO_4 _is apparently a better choice than phenytoin for the prevention of seizure in eclampsia and severe preeclampsia, phenytoin can be considered for treatment in specific conditions such as mild preeclampsia, owing to its lower risk of convulsion. Phenytoin can be practically implicated in low- and middle-income countries where the capacity for maternal monitoring is limited, considering the potential for toxicity of MgSO_4_ when administered without intensive care. Further studies are recommended with larger sample size and different drug dosage.

## Notes:

**How to cite this article:** Khooshideh M, Ghaffarpour M, Bitarafan S. The comparison of anti-seizure and tocolytic effects of phenytoin and magnesium sulphate in the treatment of eclampsia and preeclampsia: A randomised clinical trial. Iran J Neurol 2017; 16(3): 125-9.
